# Chronic inflammatory demyelinating polyneuropathy with hypoglossal nerve involvement and inverted Beevor’s sign: case report

**DOI:** 10.1186/s12883-021-02287-5

**Published:** 2021-06-26

**Authors:** Huajian Zhao, Yiming Zheng, Lingchao Meng, Meng Yu, Wei Zhang, He Lv, Zhaoxia Wang, Hongjun Hao, Yun Yuan

**Affiliations:** 1 Neurology Department, Peking University First Hospital, No.8 Xishiku Street, Beijing, 100034 China; 2 Neurology Department, University of Chinese Academy of Sciences Shenzhen Hospital (Guangming), No. 4253 Matian Street, Shenzhen, 518000 China

**Keywords:** Chronic inflammatory demyelinating polyneuropathy, Hypoglossal neuropathy, Facial neuropathy, Beevor’s sign

## Abstract

**Background:**

Cranial nerve involvement is not commonly encountered in chronic inflammatory demyelinating polyradiculoneuropathy (CIDP); this is especially true for involvement of the hypoglossal nerve. Neither Beevor's sign nor its inverted form has previously been described in CIDP.

**Case presentation:**

A 28-year-old man presented with distal-predominant limb weakness and numbness at the age of 18. A diagnosis of CIDP was made, which was confirmed by electrodiagnostic evidence of demyelination. He responded well to intravenous immunoglobulin and glucocorticoid treatment and achieved remission for 5 years. However, the same symptoms relapsed at the age of 28 and lasted for 10 months. On examination, in addition to limb sensory impairment and muscle weakness, mild bilateral facial paresis, tongue atrophy and fasciculations, and inverted Beevor's sign were also observed. A brief literature review of cranial nerve involvements in CIDP and Beevor's sign or its inverted form were also performed.

**Conclusions:**

Cranial nerves may be affected in patients with CIDP. Facial palsy is most frequently present, while hypoglossal nerve involvement is rare. Inverted Beevor's sign can appear in CIDP patients.

**Supplementary Information:**

The online version contains supplementary material available at 10.1186/s12883-021-02287-5.

## Background

Chronic inflammatory demyelinating polyneuropathy (CIDP) is a rare, acquired, immune-mediated neuropathy that affects the peripheral nerves and nerve roots. It is characterized by a relapsing–remitting or progressive course and evidence of demyelination [1]. Typical CIDP is a symmetric sensorimotor polyneuropathy that causes both proximal and distal limb weakness, sensory impairment, and areflexia. Cranial nerve involvement is not commonly encountered; this is especially true for the involvement of the hypoglossal nerve [2, 3]. Beevor's sign is an upward umbilicus deviation when a supine patient tries to sit up, and indicates predominant weakness of the lower rectus abdominis muscle. It can also be present in an “inverted” form [4]. Beevor's sign or its inverted form is mainly present in myopathies, such as facioscapulohumeral muscular dystrophy, but to our knowledge, it has never been described in CIDP [5]. In the present report, we describe uncommon findings in a patient with CIDP, cranial nerve involvement, and inverted Beevor's sign, and report the results of a brief literature review. Literature search with relevant keywords (including cranial nerve and CIDP, or Beevor's sign) was conducted in the PubMed databases up to October 2020.

## Case presentation

A 28-year-old man presented with a relapsing–remitting course of distal-predominant limb weakness and numbness for 10 years. Ten years earlier, he had gradually progressive weakness and numbness in the distal limbs. He was unable to write, button his clothes, or open jars, but had no difficulty in climbing stairs or rising from a seated position. He was treated with intravenous dexamethasone and subsequent oral prednisolone for 1 month in another hospital, although the specific dosage was unknown. His symptoms disappeared after treatment. However, 7 years ago, the same symptoms relapsed and were gradually progressive. He presented at our hospital because of persistence of the aforementioned symptoms. He was treated with intravenous immunoglobulin at 0.4 g/kg/day for 5 days, and intravenous methylprednisolone at 500 mg per day for 3 days, followed by 240 mg per day for 1 day and 120 mg per day for 1 day. Subsequently, he received 55 mg of oral prednisolone for 1 month, after which it was gradually tapered off. He also took cyclosporine at 50 mg twice per day. In addition, he was subjected to another two 5-day courses of intravenous immunoglobulin, at 6 years ago and 5 years ago. He began to gradually recover after treatment and achieved remission, and he stopped all treatment 5 years ago. However, his symptoms relapsed 10 months ago, with gradually progressive asymmetrical weakness and numbness in his distal arms and legs. His left upper limb was most affected. He had no known comorbid conditions and no relatives with a similar condition.

**Fig. 1:**
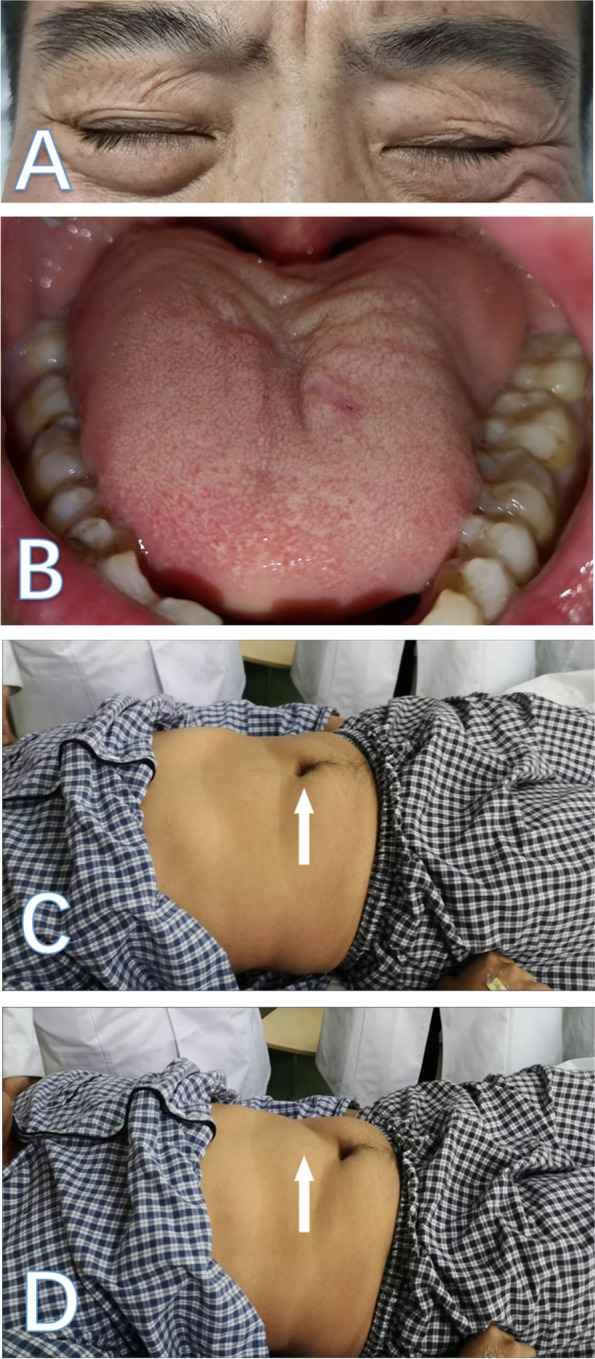
Cranial nerve involvement and inverted Beevor's sign in the patient. The patient was unable to bury the eyelashes during forced eyelid closure, indicating facial muscle weakness (**A**). His tongue showed marked asymmetrical atrophy (**B**). A downward movement of his umbilicus while attempting to stand from a recumbent position (**C** and **D**)


On neurological examination, the patient had mild bilateral facial paresis, asymmetric atrophy of the tongue muscle, and tongue fasciculations (Fig. 1, A and B, and Video 1). No dysarthria or dysphagia was observed. Pinprick and vibration sensations under the wrists and ankles were significantly decreased, while sensation of the trunk was normal. No marked atrophy of the limb muscles was observed. The arms and legs were asymmetrically weak, with a Medical Research Council scale strength of 1–4, and predominant involvement of the upper distal extremities (Supplementary Materials Table 1). Tendon reflexes including bilateral biceps, triceps, brachioradialis, knee and ankle tendon reflexes were absent, and the Babinski sign was negative. An inverted Beevor's sign was also observed, characterized by a downward movement of the patient’s umbilicus while attempting to stand from a recumbent position (Fig. 1, C and D, and Video 2).

**Additional file 1: Video 1.**


**Additional file 2: Video 2.**

Laboratory evaluations, including a complete blood count, erythrocyte sedimentation rate, C-reactive protein, antinuclear antibodies, anti-neutrophil cytoplasmic antibody, fasting serum glucose, thyroid function studies, serum and urine protein electrophoresis and immunofixation, and angiotensin-converting enzyme, were all unremarkable. Anti-ganglioside antibodies, myelin-associated glycoprotein, neurofascin 155 and 186, and contactin 1 were all negative. In addition, cerebrospinal fluid analysis revealed a normal protein content, cell count, and glucose level. Electrodiagnostic testing revealed severe, diffuse, asymmetric sensorimotor polyneuropathy that fulfilled the criteria for primary demyelination in CIDP. This included prolonged distal motor latencies, delay and disappearance of F waves, and conduction block in the radial and ulnar nerves (Table 1). The nerve ultrasonography showed segmental hypertrophy in the bilateral median and ulnar nerves (Supplementary Materials Table 2). Magnetic resonance imaging of the lumbosacral plexus revealed that the L5 and S1 spinal roots were mildly enlarged (Supplementary Materials Fig. 1). A biopsy of the left sural nerve was almost normal and vasculitis was excluded.

**Table 1:** Nerve conduction velocity study of the patient

Nerves	Left			Right			
**Motor nerve**	**MCV (m/s)**	**CMAP (mV)**	**Distal latency (ms)**	**MCV (m/s)**	**CMAP (mV)**		**Distal latency (ms)**
**Median nerve**							
Wrist-APB	no response			no response			
**Ulnar nerve**							
Wrist-ADM		3.7	2.6		7.8		2.6
Below elbow-Wrist	42.9	1.2		50.4	5.9		
Above elbow-Below elbow	no response			no response			
**Radial nerve**							
Elbow-EDC		5.0	3.1		8.9		2.8
Axilla-Elbow	58.8	1.6		64.5	3.6		
**Peroneal nerve**							
Ankle-EDB	no response			no response			
CF-Down-2 cm-Tibialis anterior muscle		6.2	4.1		5.3		2.8
CF-Up-9 cm-CF-Down 2 cm	34.7	3.7		42.0	3.0		
**Tibial nerve**							
Ankle- Abd hal		3.6	6.0		2.8		4.3
Popliteal fossa-Ankle	56.1	1.0		47.9	1.7		
**Sensory nerve**	**SCV (m/s)**	**SNAP (μV)**	**Distal latency (ms)**	**SCV (m/s)**	**SNAP (μV)**		**Distal latency (ms)**
**Median nerve**	38.9	2.0	4.1	no response			
**Ulnar nerve**	38.1	2.2	2.9	no response			
**Radial nerve**	42.9	4.7	1.8	54.5	6.1	1.7	
**Sural nerve**	After biopsy			61.8	14		1.7
**Posterior tibial nerve**	no response			no response			
**F wave conduction velocity**	44 m/s(Ulnar nerve); Occurrence rate of 5%						

Abbreviations: *MCV* Motor conduction velocity, *CMAP* Compound motor action potential, *SCV* Sensory conduction velocity, *SNAP* Sensory nerve action potential, *APB* Abductor pollicis brevis, *ADM* Abductor digiti minimi, *EDC* Extensor digitorum communis, *EDB* Extensor digitorum brevis, *CF* Capitulum fibula, *Abd hal* Abductor hallucis

The patient was diagnosed with CIDP, and was again treated with intravenous immunoglobulin at 0.4 g/kg/day for 5 days, intravenous methylprednisolone at 500 mg per day for 3 days, and then oral prednisolone at 60 mg/day for 1 month before it was gradually tapered off. The patient was followed up by telephone. After 3 months of treatment, his numbness was completely relieved, his limb strength was recovered about 70%, he had no difficulty in daily life, and he returned to work.

## Discussion and conclusions

In the current report, we present a case with chronically recurrent limb weakness and sensory dysfunction as well as cranial nerve involvement. The patient’s tendon reflexes were absent in all extremities. His nerve conduction velocity study results met the electrodiagnostic criteria for CIDP, and he responded well to immunomodulatory treatment. Other causes were excluded by extensive clinical and laboratory evaluations. According to the European Federation of Neurological Societies and the Peripheral Nerve Society criteria, the diagnosis of the present patient was definite CIDP [6]. Furthermore, the upper limb predominance and asymmetric involvement in this patient indicated a diagnosis of an atypical variant of CIDP: multifocal acquired demyelinating sensory and motor neuropathy (MADSAM), also known as Lewis–Sumner syndrome [7].

One notable feature in the present case was cranial nerve involvement, which included the facial and hypoglossal nerves. Cranial neuropathy is clinically uncommon in patients with CIDP, although it has been described in several case series or reports, including in cranial nerves II to X, and XII [2, 3, 8–11] (Table 2). A review of the literature revealed a reported incidence of cranial nerve involvement in 5% to 20% of patients with CIDP. According to a retrospective study, cranial palsy is frequent in MADSAM (48%), but less frequent in typical CIDP (11%) and distal acquired demyelinating symmetric (11%) [3]. Facial paralysis is the most common involved, followed by bulbar involvement and oculomotor nerve paralysis [3, 8–11]. Typical CIDP patients usually have bilateral cranial nerve involvement, while MADSAM patients frequently have unilateral paralysis [3]. However, reports of hypoglossal neuropathy in CIDP are especially rare [2, 12]. The present case adds to the few reported cases that indicate the presence of hypoglossal neuropathy in CIDP. Even if tongue fasciculation and atrophy is observed, CIDP should be considered because it is a treatable disorder. Tests should include nerve conduction studies to evaluate for conduction block, demyelination, and sensory nerve involvement, to distinguish this condition from motor neuron syndromes.

**Table 2:** Literature review of cranial nerve involvement in CIDP

Cranial nerve involved	Shibuya et al 132 cases [3]	Rotta et al 87 cases [8]	Barohn et al 60 cases [9]	McCombe et al 92 cases [10]	Gorson et al 67 cases [11]
I	0	0	0	0	
II	1 (1%)	0	0	0	
Ophthalmoplegia (III, IV or VI)	7 (5%)	3 (3%)	2(3%)	4(4%)	2 (3%)
V	4 (3%)	0	0	0	
VII	11 (8%)	2 (2%)	8(13%)	14(15%)	7 (10%)
VIII	2 (2%)	0	0	0	
IX, X	12 (9%)	1 (1%)	0	6(6%)	4 (6%)
XII	0	0	0	0	
Total	26(20%)	4 (5%)	10(17%)	15(16%)	

Another notable feature in the present case was the presence of an inverted Beevor's sign, which has not been reported previously in CIDP. Beevor's sign, which is an upward movement of the umbilicus with truncal flexion from a supine position, derives its name from Dr. Charles Edward Beevor. It first appeared in his 1898 textbook "Diseases of the Nervous System: A Handbook for Students and Practitioners", and was used to indicate a spinal cord lesion between the levels of T10 and T12 [4, 5, 13]. Several publications have reported this sign in other neurological disorders, but not in spinal cord lesions. It is diagnostic for facioscapulohumeral muscular dystrophy, especially in typical cases, with a specificity of 92.9% to 100% and a sensitivity of 53.6% to 95.0% [14–16]. Beevor's sign can also be present in the following conditions: GNE myopathy (observed in nearly 90% of patients) [17], late-onset Pompe disease [16, 18], tubular aggregate myopathy [15], myotonic dystrophy type 1 [16], myopathy with *TCAP* mutations [19], sporadic inclusion body myositis [20, 21], amyotrophic lateral sclerosis [22], and acute disseminated encephalomyelitis associated with demyelinating polyneuropathy [23]. Less frequently, Beevor's sign can also be present in an “inverted” form, namely as the inverted Beevor's sign, where the umbilicus moves downward because of upper rectus abdominis weakness. The inverted Beevor's sign has previously been reported in facioscapulohumeral muscular dystrophy [16, 24] and in Dr. Beevor's myopathic patient [4]. The involvement of nerve roots and extraforaminal segments of nerves in thoracic segments, as well as intercostal nerves bilaterally, has been reported in CIDP [25]. Therefore, the inverted Beevor's sign in our patient may be caused by the involvement of thoracic peripheral nerves.

In conclusion, we described a rare case of CIDP presenting with cranial nerve involvement and inverted Beevor's sign. Cranial nerves may be affected in patients with CIDP. Facial palsy is most frequently present, while hypoglossal nerve involvement is rarely reported. CIDP should be considered in patients who present with cranial nerve involvement. To our knowledge, this is the first report of the inverted Beevor's sign in CIDP. Neurologists should therefore pay attention to the movement of the umbilicus on neck flexion during neurological examinations in neuromuscular disorders.

## Supplementary Information

**Additional file 3: Table 1**. Muscle strength evaluation with Medical Research Council scale. **Table 2**. The nerve ultrasonography of the patient. **Figure 1**. The magnetic resonance imaging of the lumbosacral plexus of the patient.

## Data Availability

The datasets used and/or analyzed during the current study are available from the corresponding author on reasonable request.

## Abbreviations

CIDP: Chronic inflammatory demyelinating polyneuropathy
MADSAM: Multifocal acquired demyelinating sensory and motor neuropathy
