# Supplementary material for: Chronic inflammatory demyelinating polyneuropathy with hypoglossal nerve involvement and inverted Beevor’s sign: case report
Source: BMC Neurol. 2021 Jun 26;21:244. doi: 10.1186/s12883-021-02287-5 (PMC8235827; doi:10.1186/s12883-021-02287-5)
Supplement: Supplementary file 3 — Additional file 3: Table 1. Muscle strength evaluation with Medical Research Council scale. Table 2. The nerve ultrasonography of the patient. Figure 1. The magnetic resonance imaging of the lumbosacral plexus of the patient. [file 12883_2021_2287_MOESM3_ESM.docx]

**Supplementary materials**

**Table 1. Muscle strength evaluation with Medical Research Council scale**

|  | Left | Right |
| --- | --- | --- |
| Shoulder abduction | 5 | 5 |
| Elbow flexion | 4 | 5 |
| Elbow extension | 4 | 5 |
| Wrist extension | 3 | 4 |
| Wrist flexion | 3 | 4 |
| Finger abduction | 3 | 3 |
| Finger adduction | 1 | 2 |
| Hip flexion | 5 | 5 |
| Knee extension | 5 | 5 |
| Knee flexion | 5 | 5 |
| Ankle dorsiflexion | 5 | 5 |
| Ankle plantar flexion | 5 | 5 |
| Dorsiflexion of the great toe | 4 | 4 |
| Plantar flexion of the great toe | 5 | 5 |

**Table 2. The nerve ultrasonography of the patient**

|  | Cross-sectional areas -Left (mm^2^) | Cross-sectional areas -Right (mm^2^) | Cross-sectional areas in Healthy control* |
| --- | --- | --- | --- |
| Median nerve at | | | |
| Upper arm | **13.8** | **13.7** | 9.1±1.9 |
| Elbow | **15.3** | **13.8** | 9.1±2.0 |
| Forearm | 7.6 | **9.7** | 7.3±1.4 |
| Wrist | **11.6** | **16.5** | 8.3±1.7 |
| Ulnar nerve at | | | |
| Elbow | **9.3** | **10.1** | 8.2±2.0 |
| Forearm | 7.2 | 7.1 | 5.8±1.4 |
| Wrist | 3.9 | 3.7 | 5.0±1.7 |
| Sciatic nerve  (1/3 of mid-lower part of the thigh) | 46.7 | 48.6 | 54.2±15.7 |
| Tibial nerve  (popliteal fossa) | 25.1 | 35.4 | 32.4±7.6 |
| Common peroneal nerve  (capitulum fibulae) | 7.2 | 8.8 | 11.5±3.8 |
| Sural nerve  (lower leg near lateral malleolus) | After biopsy | 2.4 | 5.2±1.3 |

*The data of cross-sectional areas in healthy control were cited from our other article (Du, Kang et al. “Nerve Ultrasound Comparison Between Transthyretin Familial Amyloid Polyneuropathy and Chronic Inflammatory Demyelinating Polyneuropathy.” Frontiers in neurology vol. 12 632096. 26 Feb. 2021).

**Figure 1. The magnetic resonance imaging of the lumbosacral plexus of the patient**

**
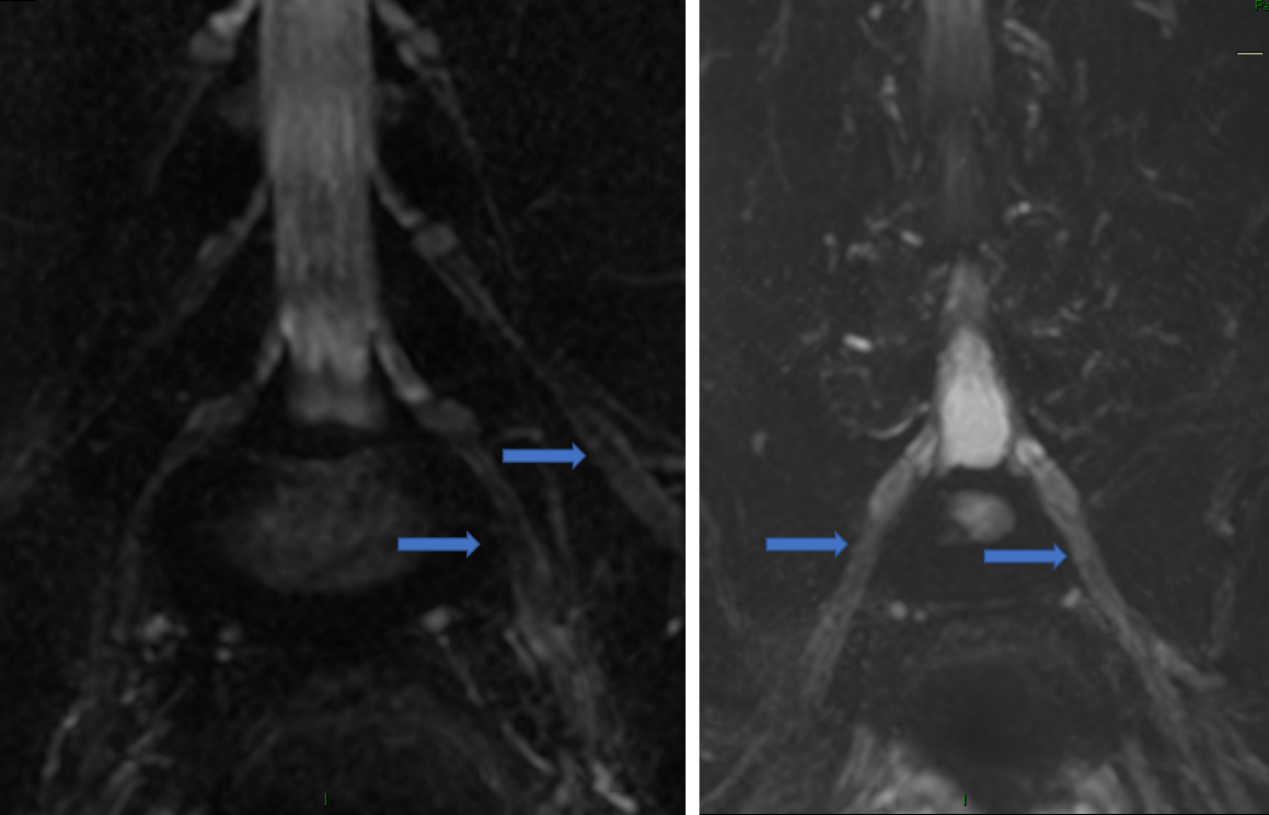
**

The magnetic resonance imaging of the lumbosacral plexus of the patient revealed that the L5 and S1 spinal roots were mildly enlarged as showed by arrows.
